# Information Extraction From the GDELT Database to Analyse EU Sovereign Bond Markets

**DOI:** 10.1007/978-3-030-66981-2_5

**Published:** 2021-01-15

**Authors:** Sergio Consoli, Luca Tiozzo Pezzoli, Elisa Tosetti

**Affiliations:** 8 UniCredit, Milan, Italy; 9 UniCredit, Rome, Italy; 10 UniCredit, Milan, Italy; 11 UniCredit, Rome, Italy; 12 ENEA Portici Research Center, Portici, Italy; 13 UniCredit, Rome, Italy; 14 Joint Research Centre, Directorate A-Strategy, Work Programme and Resources, Scientific Development Unit, European Commission, Via E. Fermi 2749, 21027 Ispra, VA Italy; 15 Department of Management, Universitá Ca’ Foscari Venezia, Cannaregio 873, 30121 Fondamenta San Giobbe, Venice Italy

**Keywords:** Big data, Government yield spread, GDELT, Machine learning, Features engineering

## Abstract

In this contribution we provide an overview of a currently on-going project related to the development of a methodology for building economic and financial indicators capturing investor’s emotions and topics popularity which are useful to analyse the sovereign bond markets of countries in the EU.These alternative indicators are obtained from the Global Data on Events, Location, and Tone (GDELT) database, which is a real-time, open-source, large-scale repository of global human society for open research which monitors worlds broadcast, print, and web news, creating a free open platform for computing on the entire world’s media. After providing an overview of the method under development, some preliminary findings related to the use case of Italy are also given. The use case reveals initial good performance of our methodology for the forecasting of the Italian sovereign bond market using the information extracted from GDELT and a deep Long Short-Term Memory Network opportunely trained and validated with a rolling window approach to best accounting for non-linearities in the data.

## Introduction and Preliminaries

Economic and fiscal policies conceived by international organizations, governments, and central banks heavily depend on economic forecasts, in particular during times of economic turmoil like the one we have recently experienced with the COVID-19 virus spreading world-wide [30]. The accuracy of economic forecasting and nowcasting models is however still problematic since modern economies are subject to numerous shocks that make the forecasting and nowcasting tasks extremely hard, both in the short and in the medium-long run. In this context, the use of recent *Big Data* technologies for improving forecasting and nowcasting for several types of economic and financial applications has high potentials. In a currently on-going project we are designing a methodology to extract alternative economic and financial indicators capturing investor’s emotions, topics popularity, and economic and political events, from the *Global Database of Events, Language and Tone (GDELT)*^1^ [17], a novel big database of news information. GDELT is a real-time, open-source, large-scale repository of global human society for open research which monitors worlds broadcast, print, and web news. The news-based economic and financial indicators extracted from GDELT can be used as alternative features to enrich forecasting and nowcasting models for the analysis of the sovereign bond markets of countries in the EU.

The very large dimensions of GDELT make unfeasible the use of any relational database and require ad-hoc big data management solutions to perform any kind of analysis in reasonable time. In our case, after GDELT data are crawled from the Web by means of custom REST APIs^2^, we use *Elasticsearch* [13, 24] to host and interact with the data. Elasticsearch is a popular and efficient NO-SQL big data management system whose search engine relies on the Lucene library^3^ to efficiently transform, store, and query the data.

After GDELT data are stored into our Elasticsearch infrastructure, a feature selection procedure selects the variables having higher forecasting potentials to analyse the sovereign bond market of the EU country under study. The selected variables capture, among others, investor’s emotions, economic and political events, and popularity of news thematics for that country. These additional variables are included into economic forecasting and nowcasting models with the goal of improving their performance. In current research we are experimenting different models, ranging from traditional economic models to novel machine learning approaches, like Gradient Boosting Machines and Recurrent Neural Networks (RNNs), which have been shown to be successful in various forecasting problems in Economics and Finance (see e.g. [4, 6–8, 16, 18, 29] among others).

## Related Work

The recent surge in the government yield spreads in countries within the Euro area has originated an intense debate about the determinants and sources of risk of sovereign spreads. Traditionally, factors such as the creditworthiness, the sovereign bond liquidity risk, and global risk aversion have been identified as the main factors having an impact on government yield spreads [3, 22]. However, a recent literature has pointed at the important role of financial investor’s sentiment in anticipating interest rates dynamics [19, 26]. An early paper that has used a sentiment variable calculated on news articles from the Wall Street Journal is [26]. In this work it is showed that high levels of pessimism are a relevant predictor of convergence of the stock prices towards their fundamental values. Other recent works in finance exist on the use of emotions extracted from social media, financial microblogs, and news to improve predictions of the stock market (e.g. [1, 9]). In the macroeconomics literature, [14] has looked at the informational content of the Federal Reserve statements and the guidance that these statements provide about the future evolution of monetary policy. Other papers ([27, 28] and [25] among others) have used Latent Dirichlet allocation (LDA) to classify articles in topics and to extract a signal with predictive power for measures of economic activity, such as GDP, unemployment and inflation [12]. These results, among others, have shown the high potentials of the information extracted from news variables on monitoring and improving the forecasts of the business cycle [9].

Machine learning approaches in the existing literature for controlling financial indexes measuring credit risk, liquidity risk and risk aversion include the works in [3, 5, 10, 11, 20], among others. Several efforts to make machine learning models accepted within the economic modeling space have increased exponentially in recent years (see e.g.. [4, 6–8, 16, 18, 29] among others).

## GDELT Data

GDELT analyses over 88 million articles a year and more than 150,000 news outlets. Its dimension is around 8 TB, growing 2TB each year [17]. For our study we rely on the “Global Knowledge Graph (GKG)” repository of GDELT, which captures people, organizations, quotes, locations, themes, and emotions associated with events happening in print and web news across the world in more than 65 languages and translated in English. Themes are mapped into commonly used practitioners’ topical taxonomies, such as the “World Bank (WB) Topical Ontology”^4^. GDELT also measures thousands of emotional dimensions expressed by means of, e.g., the “Harvard IV-4 Psychosocial Dictionary”^5^, the “WordNet-Affect dictionary”^6^, and the “Loughran and McDonald Sentiment Word Lists dictionary”^7^, among others. For our application we use the GDELT GKG fields from the World Bank Topical Ontology (i.e. WB themes), all emotional dimensions (GCAM), and the name of the journal outlets.

The huge number of unstructured documents coming from GDELT are re-engineered and stored on an ad-hoc Elasticsearch infrastructure [13, 24]. Elasticsearch is a popular and efficient document-store built on the Apache Lucene search library^8^ and providing real-time search and analytics for different types of complex data structures, like text, numerical data, or geospatial data, that have been serialized as JSON documents. Elasticsearch can efficiently store and index data in a way that supports fast searches, allowing data retrieval and aggregate information functionalities via simple REST APIs to discover trends and patterns in the stored data.

## Feature Selection

We use the available World Bank Topical Ontology to understand the primary focus (theme) of each article and select the relevant news whose main themes are related to events concerning bond market investors. Hence, we select only articles such that the topics extracted by GDELT fall into one of the following WB themes of interest: *Macroeconomic Vulnerability and Debt*, and *Macroeconomic and Structural Policies*. To make sure that the main focus of the article is one of the selected WB topics, we retain only news that contain in their text at least three keywords belonging to these themes. The aim is to select news that focus on topics relevant to the bond market, while excluding news that only briefly report macroeconomic, debt and structural policies issues. We consider only articles that are at least 100 words long. From the large amount of information selected, we construct features counting the total number of words belonging to all WB themes and GCAMs detected each day. We also create the variables “Number of mentions“ denoting the word count of each location mentioned in the selected news. We further filter the data by using domain knowledge to retain a subset of GCAM dictionaries that qualitatively may have potentials to our analysis. Then we retain only the variables having a standard deviation calculated over the full sample greater than 5 words and allowing a 10$\%$ of missing values on the total number of days. Finally we perform a correlation analysis across the selected variables, normalized by number of daily articles. If the correlation between any two features is above 80$\%$ we give preference to the variable with less missing values, while if the number of missing values is identical and the two variables belong to the same category (i.e. both are themes or GCAMs), we randomly pick one of them. Finally, if the number of missing values is identical but the two variables belong to the same category, we consider the following order of priority: GCAM, WB themes, GDELT themes, locations.

## Preliminary Results

Here we show some preliminary results on the application of the described methodology for the use case of Italy. The main objective of this empirical exercise is to assess the predictive power of GDELT selected features for the forecasting of the Italian sovereign bond market.

**Fig. 1.**
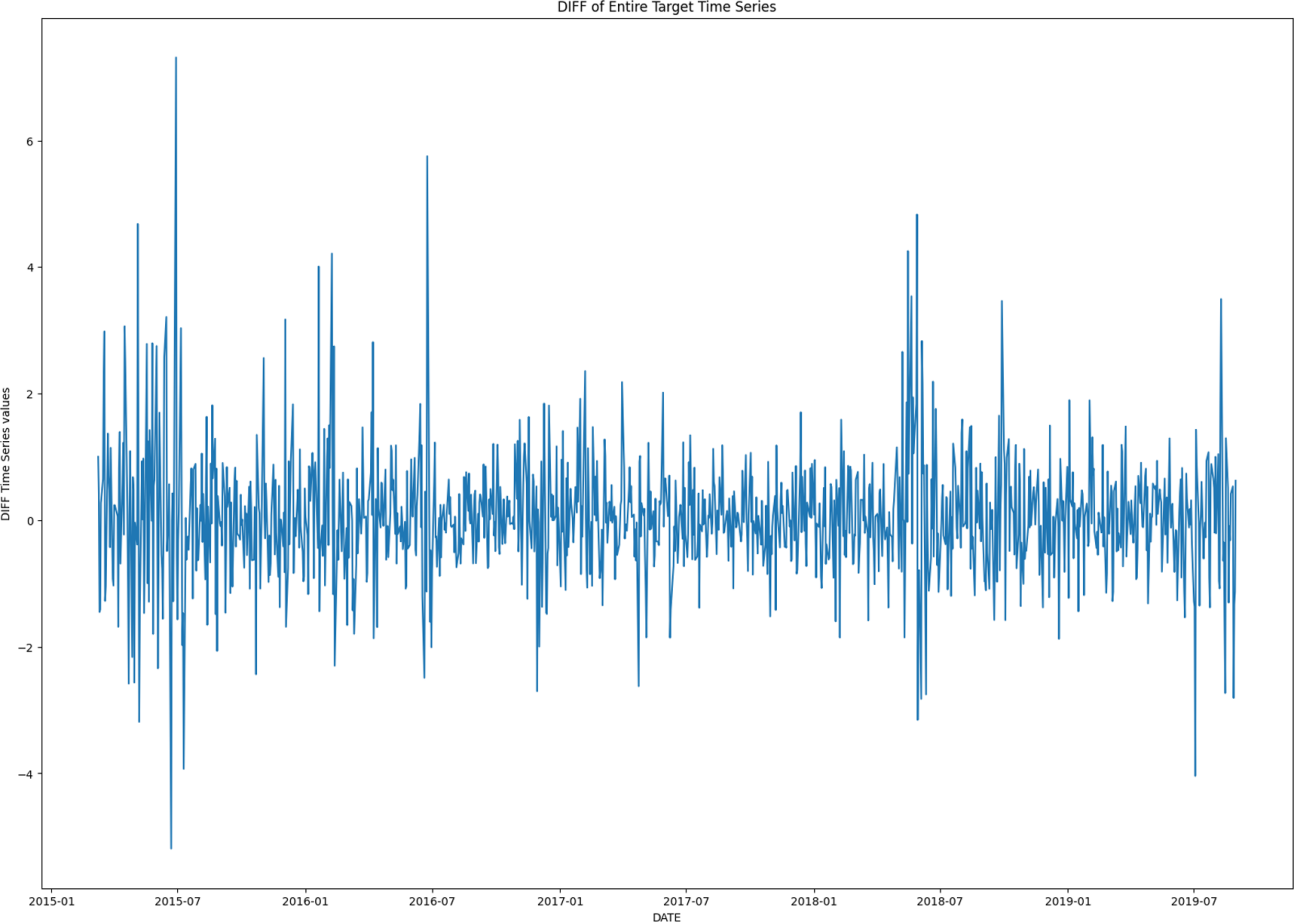
Log-differences of the sovereign spread for Italy against Germany as the difference between the Italian 10 year maturity bond yield minus the German counterpart.

We have extracted data from Bloomberg on the term-structure of government bond yields for Italy over the period 2 March 2015 to 31 August 2019. We have calculated the sovereign spread for Italy against Germany as the difference between the Italian 10 year maturity bond yield minus the German counterpart. We have also extracted the standard level, slope and curvature factors of the term-structure using the Nelson and Siegel [23] procedure and included these classical factors into the model. Being the government bond yields a highly persistent and non-stationary process, we have considered its log-differences and obtained a stationary series of daily changes representing our prediction target, illustrated in Fig. 1. This kind of forecasting exercise is an extremely challenging task, as the target series behaves similarly to a random walk process. Missing data, related to weekends and holidays, have been dropped from the target time series, giving a final number of 468 data points.

**Fig. 2.**
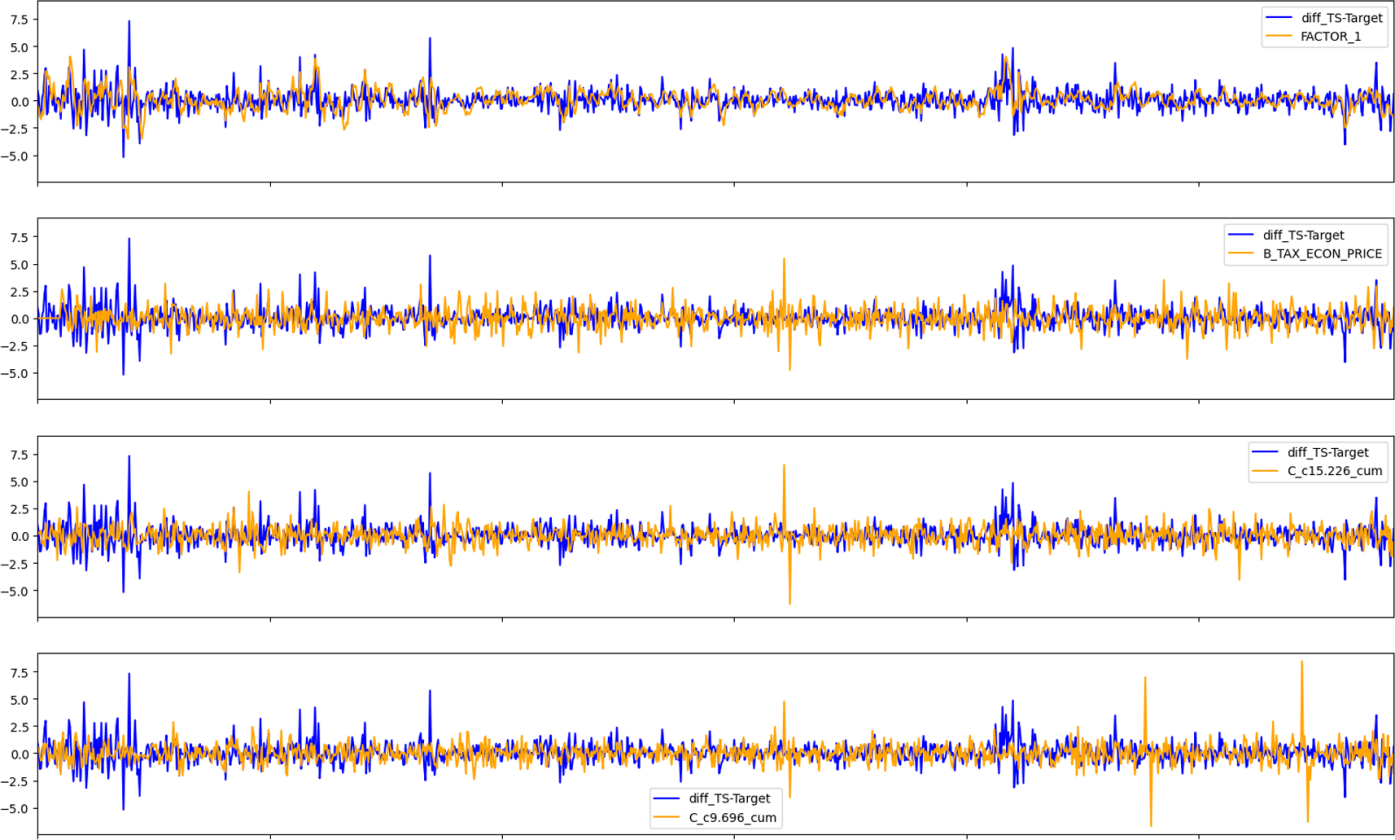
Log-differences of the sovereign spread for Italy against Germany as the difference between the Italian 10 year maturity bond yield minus the German counterpart.

For our Italian case study, we have also extracted the news information from GKG in GDELT from a set of around 20 newspapers for Italy, published over the considered period of the analysis. After this selection procedure we obtained a total of 18,986 articles, with a total of 2,978 GCAM, 1,996 Themes and 155 locations. Applying the feature selection procedure described above, we have extracted 31 dimensions of the General Inquirer Harvard IV psychosocial Dictionary, 61 dimensions of Roget’s Thesaurus, 7 dimensions of the Martindale Regressive Imagery and 3 dimensions of the Affective Norms for English Words (ANEW) dictionary. After the features engineering procedure, we have been left with a total of 45 variables, of which 9 are themes, 34 are GCAM, 2 locations. The selected topics contained WB themes such as Inflation, Government, Central Banks, Taxation and Policy, which are indeed important thematics discussed in the news when considering interest rates issues. Moreover, selected GCAM features included optimism, pessimism or arousal, which explore the emotional state of the market. Figure 2 shows the top correlated covariates with respect to the target.

Several studies in the literature have shown that during stressed periods, complex non-linear relationships among explanatory variables affect the behaviour of the output target which simple linear models are not able to capture. For this reason, in this empirical exercise we have used a deep Long Short-Term Memory Network (LSTM) [15] to best accounting for non-linearities and assessing the predictive power of the selected GDELT variables. The LSTM was implemented relying on the DeepAR model available in Gluon Time Series (GluonTS) [2]^9^, an open-source library for probabilistic time series modelling that focuses on deep learning-based approaches and interfacing Apache MXNet^10^. DeepAR is an LSTM model working into a probabilistic setting, that is, predictions are not restricted to point forecasts only, but probabilistic forecastings are produced according to a user-defined predictive distribution (in our case a student *t*-distribution was experimentally selected). For our experiment we have set experimentally to use 2 RNN layers, each having 40 LSTM cells, and used a learning rate equal to 0.001. The number of training epochs was set to 500, with training loss being the negative log-likelihood function.

We have used a robust scaling for the training variables by adopting statistics robust to the presence of outliers. That is, we have removed the median to each time series, and the data were scaled according to the interquartile range. Furthermore we have adopted a rolling window estimation technique where the first estimation sample started at the beginning of March and ended in May 2017. For each window, one step-ahead forecasts have been calculated. The whole experiment required to run few hours in parallel on 40 cores at 2.10 GHz each into an Intel(R) Xeon(R) E7 64-bit server having overall 1 TB of shared RAM.

**Fig. 3.**
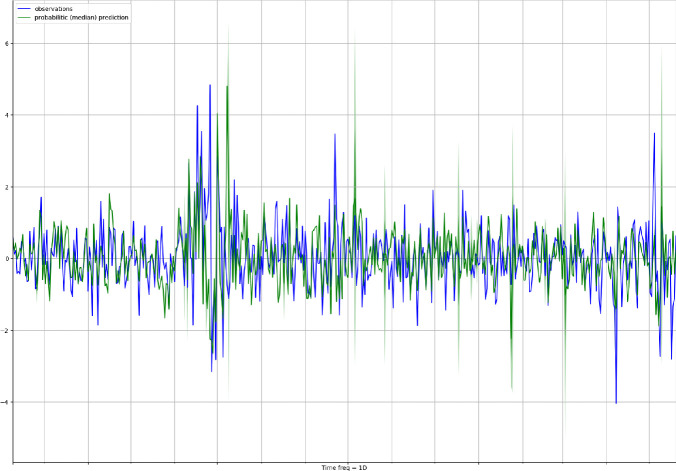
Median forecasts (green) and observations for the target series (blue) for the entire forecasting period. (Color figure online)

**Fig. 4.**
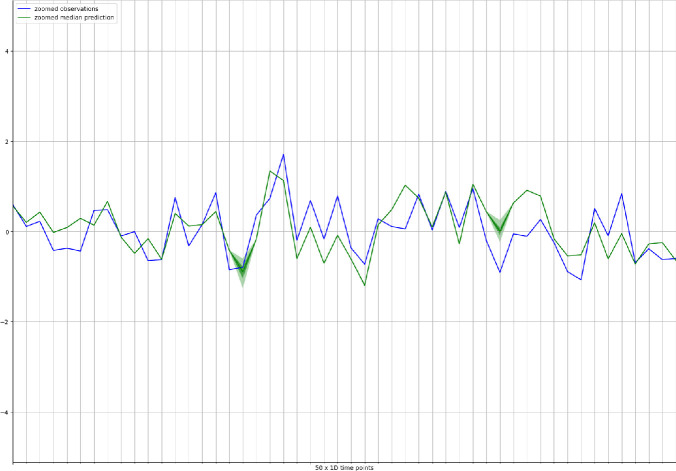
Probabilistic forecasts (green) and observations for the target series (blue) for the first 50 days in the testing period. The green continuous line shows the median of the probabilistic predictions, while the lighter green areas represents an higher confidence interval. (Color figure online)

Figure 3 shows the observations for the target time series (blue line) together with the median forecast (dark green line) and the confidence interval in lighter green. To better visualize the differences between observed and predicted time series, we have reported the same plot on a smaller time range (50 days) in Figure 4. A qualitative analysis of the figure suggests that the forecasting model does a reasonable job at capturing the variability and volatility of the time series.

**Table 1.** Forecasting results of the LSTM model in terms of MASE, sMAPE, RMSE, and wQuantileLoss error metrics.

*Metrics*	**LSTM results**	
	*In-sample*	*Out-of-sample*
MASE	0.112	0.682
sMAPE	0.130	1.148
RMSE	0.493	0.885
wQuantileLoss[0.1]	0.050	0.869
wQuantileLoss[0.3]	0.115	0.899
wQuantileLoss[0.5]	0.151	0.914
wQuantileLoss[0.7]	0.121	0.923
wQuantileLoss[0.9]	0.047	0.907

**Fig. 5.**
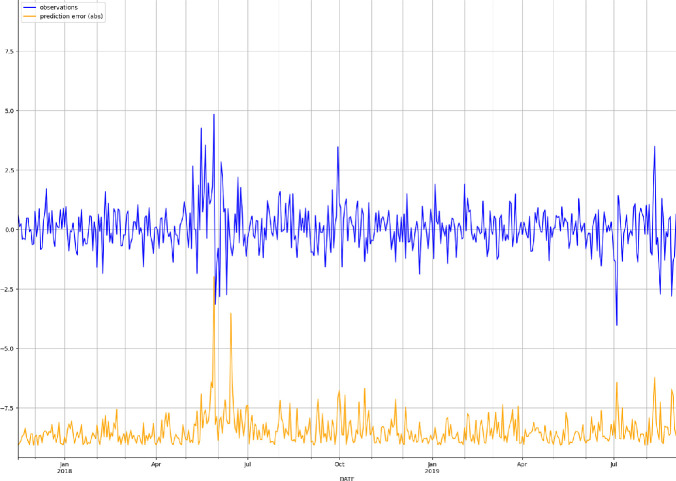
Mean absolute forecast error (MAFE) (orange) against real observations (blue). (Color figure online)

We have also computed a number of commonly used evaluation metrics [21], such as the mean absolute scaled error (MASE), the symmetric mean absolute percentage error (sMAPE), the root mean square error (RMSE), and the (weighted) quantile losses (wQuantileLoss), that is the quantile negative log-likelihood loss weighted with the density. The obtained in-sample and out-of-sample results are shown in Table 1. As expected the results worsen passing from the in-sample to the out-of-sample setting, but the gap is absolutely acceptable, confirming a good generalization capability of the trained LSTM model. The model showed higher performance at high (0.9) and low (0.1) quantiles with lower weighted quantile losses. Figure 5 illustrates the median absolute forecast error (MAFE, in orange) against the real time series observations (in blue). The performance of the model slightly worsen from the end of May to July 2018, corresponding to a period of political turmoil in Italy. Indeed, on the 29$^{th}$ of May, the Italian spread sharpely rose reaching 250 basis point. Investors where particularly worried about the possibility of anti-euro government and not confident on the formation of a stable government. From June until November 2018, a series of discussions about deficit spending engagements and possible conflicts with European fiscal rules continued to worry the markets. The spread strongly increased in October and November with values around 300 basis point. We can see this also from the performance of our model which worsen a bit in this stressed period, which however the model looks to handle quite well anyway. Since 2019, the Italian political situation started to improve and the spread smoothly declined, especially after the agreement with Brussels on budget deficit in December 2018. However, some events hit the Italian economy afterwards, such as the EU negative outlook and the European parliament elections which contributed to a temporary increase on interest rates. Our model performs quite well in this period in terms of absolute error ratios showing a good robustness.

**Fig. 6.**
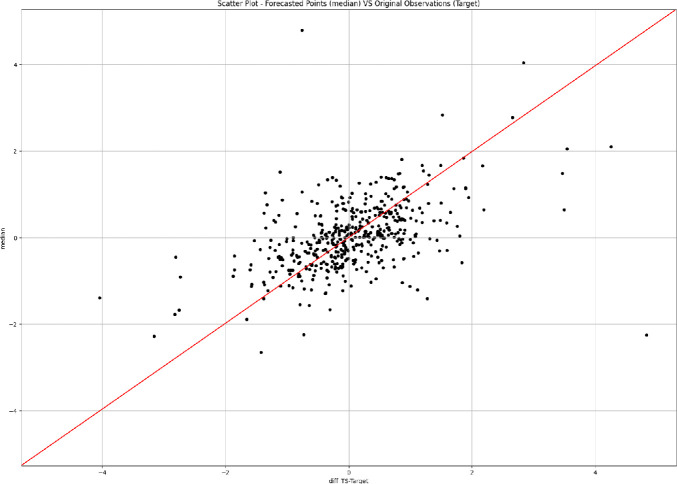
Scatter plot amongst the median out-of-sample forecasted points and the real observations.

Figure 6 shows a scatter plot amongst the median out-of-sample forecasted points and the real observations. To some degree the points in the scatter plot roughly follow the diagonal, showing a fine correlation among the forecasted points and the real observations, and suggesting good quality of the forecasting results. This is also confirmed by the acceptable value of 0.23 computed for the R-squared metrics on the out-of-sample median forecasts for such a challenging prediction exercise. This value of the R-squared measure indicates that the LSTM model explains a quite ample variability of the response data around its median, suggesting a certain degree of closeness among the forecasted data and the real observations.

## Conclusion and Overlook

In this contribution we have presented our work-in-progress related to the development of a methodology for building alternative economic and financial indicators capturing investor’s emotions and topics popularity from GDELT, the Global Data on Events, Location, and Tone database, a free open platform containing real-time worlds broadcast, print, and web news. The currently on-going project in which this work is developed is aimed at producing improved forecasting methods to analyse the sovereign bond markets of countries in the EU. We have reported some preliminary results on the application of this methodology for predicting the Italian sovereign bond market. This use case reveals initial good performance of the methodology, suggesting the validity of the approach. Using the information extracted from the Italian news media contained in GDELT combined with a deep Long Short-Term Memory Network opportunely trained and validated with a rolling window approach, we have been able to obtain quite good forecasting results.

This work represents one of the first to study the behaviour of government yield spreads and financial portfolio decisions in the presence of classical yield curve factors and information extracted from news. We believe that these new measures are able to capture and predict changes in interest rates dynamics especially in period of turmoil. Overall, the paper shows how to use a large scale database as GDELT to derive financial indicators in order to capture future intentions of agents in sovereign bond markets.

Certainly more research is still needed to be exploited in the directions of the presented work. First we will try to improve the performance of the implemented DeepAR model by tweaking architecture and optimizing the hyperparameters of the LSTM model. Furthermore, in current research we are experimenting other different prediction models, ranging from traditional economic methods to other novel machine learning approaches, including Gradient Boosting Machines and neural forecasting methods. In a future extended version of the paper we will compare and thoroughly analyze the performance of these methods to better exploit the non-linear effects of the dependent variables. Interpretability of the implemented machine learning models by using, e.g., computed Shapley values, will be an important object of future investigation in order to finely assess the contributions of the different covariates in the models predictions.

## Footnotes

1 GDELT website: https://blog.gdeltproject.org/.

2 See https://blog.gdeltproject.org/gdelt-2-0-our-global-world-in-realtime/.

3 https://lucene.apache.org/.

4 https://vocabulary.worldbank.org/taxonomy.html.

5 Harvard IV-4 Psychosocial Dictionary: http://www.wjh.harvard.edu/~inquirer/homecat.htm.

6 WordNet-Affect dictionary: http://wndomains.fbk.eu/wnaffect.html.

7 Loughran and McDonald Sentiment Word Lists: https://sraf.nd.edu/textual-analysis/resources/.

8 https://lucene.apache.org/.

9 Available at: https://gluon-ts.mxnet.io/#gluonts-probabilistic-time-series-modeling.

10 Available at: https://mxnet.apache.org/.
